# Prevalence of anti-hepatitis C antibodies and its co-infection with HIV in rural Cameroon

**DOI:** 10.1186/s13104-018-3566-4

**Published:** 2018-07-11

**Authors:** Valirie Ndip Agbor, Claude Tayou Tagny, Jules-Bertrand Kenmegne, Bih Awazi, Charlotte Ngansop, Dora Mbanya, Nicaise Ndembi

**Affiliations:** 1 Faculty of Medicine and Biomedical Sciences (FMBS), The University of Yaoundé I, Yaoundé, Cameroon; 2 Yaoundé University Teaching Hospital (YUTH), Yaoundé, Cameroon; 3 Society for Women and AIDS in Africa (SWAA), Yaoundé, Cameroon; 4 Institute of Human Virology Nigeria, Abuja, Nigeria

**Keywords:** Hepatitis C virus, Risk factor, Human immunodeficiency virus, Co-infection, Rural Cameroon

## Abstract

**Objective:**

To evaluate the prevalence of the co-infection between the human immunodeficiency virus (HIV) and hepatitis C virus (HCV), and the prevalence of factors associated with HCV transmission in a rural Cameroonian community.

**Results:**

The mean age of the 174 participants included in the study was 30.3 (standard deviation = 13.26) years (age range 12–77 years). the prevalence of HCV/HIV co-infection was 1.7% [95% confidence interval (CI) 1.1–5.9]. The prevalence of HCV and HIV were 6.3% (95% CI 2.9–10.3) and 6.9 (95% CI 5.2–11.3), respectively. Histories of scarification (62.1%), multiple sex partners (31.0%) and sexually transmitted diseases (66.1%) were the most common risk factors of HCV transmission in this study.

## Introduction

Both hepatitis C virus (HCV) and the human immunodeficiency virus (HIV) are major public health concerns worldwide due to their high prevalences, morbidity and mortality. This is aggravated by the co-infection with both viruses, as one accelerates the course of the other [1–4].

The hepatitis C virus is a major cause of chronic liver disease, and a leading cause of non-AIDS related death among persons infected with HIV in sub-Saharan Africa [5–7]. Since the introduction of highly active antiretroviral therapy, a decline in the mortality in HIV-infected persons from opportunistic infections has been observed; in contrast, there has been a significant increase in morbidity and mortality of HIV-infected persons related to HCV infection [2, 5].

Globally, 20–30% of the 34 million persons living with HIV/AIDS are co-infected with HCV [8]. According to WHO, Africa bears the highest prevalence of chronic HCV of 5.3% [9]. The prevalence of chronic hepatitis C in Cameroon is estimated to about 13% [10]. The prevalence of HCV in Cameroon varies widely between 12.5 and 23.9% depending on the study population [11–13]. A national prevalence of HIV of 4.3% was observed in 2011, with the general population of southern Cameroon displaying the highest prevalence of HIV of 10.6% [14].

The prevalence of HCV/HIV co-infection varies between 0.0 and 7.2% among the different Cameroonian population [15–17]. Only a single study has evaluated the prevalence of HCV and HIV co-infection among the rural population [15]. With the aim of contributing to the body of knowledge on this area in Cameroon, we carried out this study to evaluate the magnitude of HCV/HIV co-infection in a rural community in the South Region of Cameroon.

## Main text

### Study design, duration and setting

This was a population-based descriptive cross-sectional study conducted from November 2014 and April 2015. Participants were consecutively recruited from Abang Minko’o; a rural community of about 4000 inhabitants in the South Region of Cameroon, along its border with Gabon. All consenting participants aged ≥ 12 years, who were resident in Abang Minko’o for at least 3 months prior to our study were included.

Serological testing was done in the field sites at Abang Minko’o and confirmed in the haematology laboratory of the Yaoundé University Teaching Hospital (YUTH) and the virology laboratory of the Centre Pasteur du Cameroun (CPC)—Yaoundé.

### Data collection and management

After informed consent was obtained, data for each participant was collected unto a pre-structured questionnaire in a private setting and a code was attributed to each participant. In order to describe the seroprevalence of HCV in our study population, data was obtained on the sociodemographic parameter (age, gender, marital status and occupation) of the study participants. Data on the following risk factors for hepatitis C transmission were collected: parenteral risks (blood transfusion and history of invasive procedures such as: minor surgery, dilatation and curettage, tooth extraction and intravenous drug use), sexual behaviour (number of lifetime sexual partners and history of sexually transmitted disease) and community acquired risk factors (scarification and tattooing in unsanitary facilities). Multiple sex partners were defined as at least three lifetime sex partners.

Five millilitres of blood was collected by venipuncture into ethylene diamine tetraacetate—containing tubes, each carrying a participant code, respecting aseptic rules. Part of the sample was used to carry out the screening tests for HIV and HCV, and the rest stored in an insulated box containing ice packs until transported to Yaoundé for further analysis.

### Serological analysis

All blood samples were screened for HIV at the recruitment site using the rapid test DETERMINE^®^ HIV1/2^a^ (Alere; Bedfordview, South Africa) and then IMMUNOCOMB^®^ II HIV 1&2 BiSpot (Organics, Yavne, Israel) for viral typing if the former was positive. All samples were retested using MUREX HIV Ag/Ab Combo^a^ (DiaSorin; Saluggia, Italy), a third generation Enzyme Linked Immunosorbent Assay (ELISA) technique, at the Haematology laboratory of the YUTH. A sample was considered positive for HIV if either the ELISA or both the ELISA and rapid tests were positive. Samples with a negative ELISA results, regardless of the outcome of the rapid tests, were considered negative.

All samples were also screened for the presence of anti-HCV antibodies using the rapid test IMMUNOCOMB^®^ II HCV (Organics, Yavne, Israel). All positive and indeterminate anti-HCV antibody samples were further retested using the Architect anti-HCV qualitative assay (Abbott, Wiesbaden, Germany), a third generation Chemiluminescent Microparticle immunoassay (CMIA), at the virology laboratory of the CPC. Samples with either a positive CMIA, or a positive rapid test and CMIA were considered positive for the presence of anti-hepatitis C antibodies.

### Statistical analysis

Data was then entered into and analysed with the statistical package for social sciences v20.0. Results were presented as means and medians with their corresponding standard deviations (SD) and interquartile ranges (IQR) for quantitative variables, while for qualitative variables, results were presented as frequencies, proportions and bar charts.

## Results

A total of 174 participants were included in this study. The female prevalence was 46.6%. The mean and median ages of our study participants was 30.12 (SD = 13.26) and 28.0 (IQR = 19.75–37.0) years, respectively. Participants ages ranged from 12 to 77 years. The 16–25 years age group was the most represented (31%). Half (52%) of the participants were single, 33% were married, meanwhile 15% of them were cohabiting, Table 1.

**Table 1:** Distribution of HCV seropositivity with socio-demographic characteristics, Abang Minko’o 2015

Characteristics	n (%) [N = 174]	HCV+ cases (%) [N = 11]
Gender		
Male	93 (53.4)	6 (54.5)
Female	81 (46.6)	5 (45.5)
Age group		
[≤ 15]	20 (11.5)	2 (18.2)
[16–25]	55 (31.6)	2 (18.2)
[26–35]	48 (27.6)	1 (9.1)
[36–45]	31 (17.8)	2 (18.2)
[46–55]	14 (8.0)	3 (27.3)
[≥ 56]	6 (3.4)	1 (9.1)
Marital status		
Married	57 (32.8)	5 (45.5)
Single	90 (51.7)	6 (54.5)
Cohabiting	27 (15.5)	0 (0.0)
Occupation		
Civil servant	19 (10.9)	2 (18.2)
Farming	29 (16.7)	1 (9.1)
Self-employed	60 (34.5)	1 (9.1)
Student	49 (28.2)	5 (45.5)
Unemployed	11 (6.3)	1 (9.1)
Others	6 (3.4)	1 (9.1)

*n* number of participants in each category/subcategory, *N* total number of participants

As shown in Table 1, eleven out of the 174 study participants tested positive for the presence of anti-HCV antibodies, giving an overall prevalence of 6.3% (95% CI 2.9–10.3), 53.4% of who were males. Also shows the distribution of anti-HCV antibodies among the different sociodemographic characteristics. Self-employed participants displayed the highest proportion of anti-HCV antibodies (34.5%), followed by students (28.2%), and then farmers (16.7%). Participants within 46–55 years age group had the highest group prevalence of anti-HCV antibodies followed by the age groups 56 years and over and 15 years and below, Table 1.

**Fig. 1:**
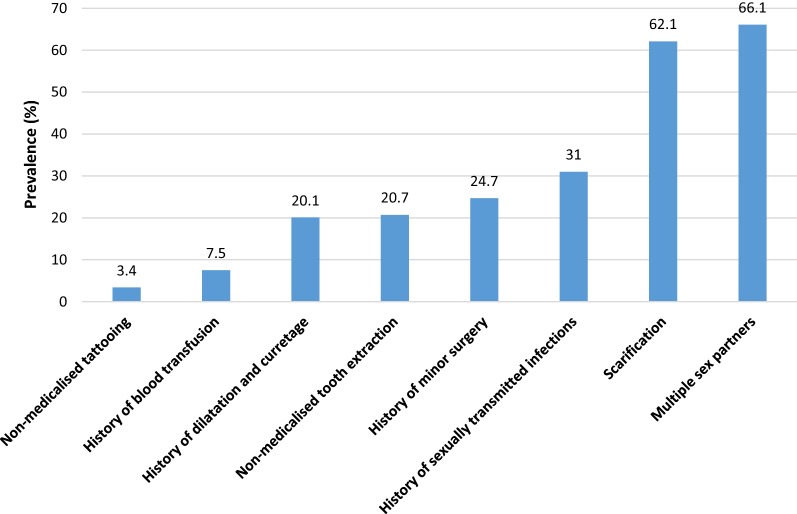
Prevalence of risk factors for hepatitis C transmission in our study population, Abang Minko 2015

Figure 1 depicts the proportion of the different risk factors of hepatitis C in our study population. Histories of multiple sexual partners (66.1%), scarification (62.1%) and sexually transmitted diseases (31.0%) were the most common risk factors in this population. Meanwhile, Histories of non-medicalised tattooing (3.4%) and blood transfusion (7.5%) were rare. No participant reported illicit intravenous drug use.

Of the 174 participants, 12 were confirmed HIV positive. Hence, an HIV prevalence of 6.9% (95% CI 5.2–11.3). A greater proportion of females (7.4%) were infected compared to males (6.5%). The 16–35 age group represented 50% of the infected cases, Table 2. The prevalence of HIV among the reproductive age group (15–49 years) was 6.4%. All samples tested positive for HIV type 1.

**Table 2:** Distribution of HIV among the different age groups, Abang Minko’o, 2015

Age group (years)	[≤ 15]	[16–25]	[26–35]	[36–45]	[46–55]	[≥ 56]	Total
HIV+ (n = 12)^a^	1	2	4	2	3	0	12
Number of participants	20	54	47	32	15	6	174
Prevalence (%)^b^	5.0	3.7	8.5	6.2	20	0	6.9
Infected cases (%)^c^	8.3	16.7	33.3	16.7	25.0	0	100.0

*HIV* human immunodeficiency virus

^a^Number of positive HIV cases

^b^Age group prevalence

^c^Percentage of infected cases

Finally, a dual infection was noted in 3 of the 174 participants giving a co-infection rate of 1.7%.

## Discussion

Herein, we report the prevalence of HCV was 6.3%. The commonest risk factors for hepatitis C transmission were histories of multiple sex partners, scarification and sexually transmitted diseases. Finally, the prevalence of HCV and HIV co-infection was 1.7%.

The seroprevalence of HCV in our study population was 6.3% is comparable to the prevalence of 6.4 and 6.9% reported by Ndumbe et al. [11] and Nerrienet et al. [18] in Cameroon. These results which are similar support the fact that Cameroon is an area where HCV is highly endemic. Also, similar prevalences of HCV; 6.0% by Hadush et al. [19] and 5.4% by Simpore et al. [20] have been reported from other sub-Sahara African countries. However, controversial prevalences have been reported by other local research works [12, 13, 18, 21]. The discrepancies in these results could be as a result of a difference in size and risk factors of HCV transmission between sample population, study methodology and geographical differences. Transmission rates vary depending on the stage of the infection, age, risk group, exposure, diagnosis status and treatment status [22]. The relatively low prevalence of blood transfusion and invasive procedures such as tattooing in non-medicalised facilities and history of dilatation and curettage, and the non-existence of intravenous drug users in our study population could explain the lower seroprevalence of HCV observed in this study. Indeed, the transmission of HCV through blood route during blood transfusion and invasive procedures has been associated with the greatest risk of hepatitis C transmission [23].

The 6.9% prevalence rate of HIV in this study is similar to the 7.4% prevalence reported in the East Region of Cameroon [15]. Other research works carried out in Cameroon have shown varying HIV prevalences. Higher prevalences of 10.7% in the Southwest Region [24], and 10.6 and 8.6% in the South Region and Yaoundé respectively [14]. Also, our finding was greater than the prevalence of HIV in the general Cameroonian population [14]. A higher prevalence of HIV infection was observed for females (7.4%) than for males (6.5%). This was consistent with the 2011 report of the national demographic and health survey conducted in Cameroon, whereby a higher prevalence of HIV was noted in females compared to males [14]. These results which are comparable support the fact that women are more vulnerable to HIV infection than men. Cultural factors, economical dependence of women on men and poor access to education and healthcare go a long way to explain why the African woman in general and Cameroonian woman in particular is susceptible to HIV [25].

The seroprevalence of HCV and HIV co-infection was 1.7%. Similar results have been reported by Laurent et al. from Cameroon [15] and other African authors; 1.6% by Hadush et al. from Ethiopia [19], 1.7% by Mboto et al. from Gambia [26], 1.86% by Onakewhor et al. from Nigeria [27] and 1.9% by Amin et al. from South Africa [28]. Even with the high prevalence of HCV and HIV, and of risky sexual behaviours, the co-infection rate between these two viruses in the general population was low. Sexual intercourse is the main route of transmission of HIV [15, 24], while blood is the main route of transmission of HCV [23]. These findings could suggest that sexual transmission of HCV is not a predominant route of transmission, but given the small sample size, this cannot be generalized. Indeed, there has been some controversies about the sexual transmission of HCV [9, 29].

## Conclusion

The prevalence of the co-infection of HCV with HIV in the study population is low. However, we noted a high seroprevalence of HCV and HIV infections. The history of multiple sex partners, scarification and sexually transmitted diseases were the most common risk factors for HCV infection in this population. Similar studies with larger sample sizes are needed in other regions to better understand the prevalence of HCV and HIV co-infection and the risk factors for HCV transmission. Public health programs aimed at educating and encouraging safe sexual behaviours in this rural community are recommended. Education on the health risks of cultural scarification practices is highly endorsed.

## Study limitations

The findings in herein should be interpreted in the context of the study limitations. Although we used very reliable methods for the diagnosis of HIV and HCV, a confirmatory assay is usually recommended. Also, our findings cannot be generalised to the South Region of Cameroon due to a small sample size and unequal representation of all age groups. Data on the risk factors of HCV in this study were subject to a recollection bias as it depended mostly on the ability of the participants to remember whether they had been exposed to the risk factor or not.

## Abbreviations

CPC: Centre Pasteur du Cameroun
HIV: human immunodeficiency virus
HCV: hepatitis C virus
YUTH: Yaoundé University Teaching Hospital
